# Impact of concomitant use of proton pump inhibitors or cardiovascular medication on survival outcomes of patients with metastatic renal cell carcinoma treated with nivolumab

**DOI:** 10.1007/s10585-025-10347-0

**Published:** 2025-08-02

**Authors:** Ondřej Fiala, Petr Hošek, Michaela Tkadlecová, Bohuslav Melichar, Anežka Zemánková, Jindřich Kopecký, Michal Vočka, Martin Matějů, Radka Lohynská, Dominika Šiková, Petr Stránský, Hana Študentová, Martina Spisarová, Hana Nováková, Peter Priester, Dominika Kryštofová, Lucie Grmelová, Tomáš Büchler, Alexandr Poprach

**Affiliations:** 1 Department of Oncology and Radiotherapeutics, Faculty of Medicine in Pilsen, Charles University, Alej Svobody 80, Pilsen, 304 60 Czech Republic; 2 Biomedical Center, Faculty of Medicine in Pilsen, Charles University, Alej Svobody 76, Pilsen, 304 60 Czech Republic; 3 Department of Oncology, Palacký University Medical School and Teaching Hospital, I.P. Pavlova 6, Olomouc, 775 20 Czech Republic; 4 Department of Oncology, University Hospital in Hradec Králové, Sokolská 581, Hradec Králové, 50005 Czech Republic; 5 Department of Oncology, First Faculty of Medicine, Charles University and General University Hospital, U Nemocnice 499/2, Prague, 128 08 Czech Republic; 6 Department of Oncology, First Faculty of Medicine, Charles University and Thomayer University Hospital, Videnska 800, Prague, 140 59 Czech Republic; 7 Department of Urology, Faculty of Medicine and University Hospital in Pilsen, Charles University, Czech Republic, Edvarda Beneše 1128/13, Pilsen, 301 00 Czech Republic; 8 Institute of Biostatistics and Analyses, Ltd, Poštovská 68/3, Brno, 602 00 Czech Republic; 9 Department of Oncology, Second Faculty of Medicine, Charles University and Motol University Hospital, V Úvalu 84, Prague, 150 06 Czech Republic; 10 Department of Comprehensive Cancer Care, Masaryk Memorial Cancer Institute, Zluty kopec 7, Brno, 656 53 Czech Republic; 11 Department of Comprehensive Cancer Care, Faculty of Medicine, Masaryk University, Kamenice 5, Brno, 625 00 Czech Republic; 12 Department of Oncology and Radiotherapeutics, Faculty of Medicine and University Hospital in Pilsen, Charles University, Czech Republic, Alej Svobody 80, Pilsen, 304 60 Czech Republic; 13 University Hospital Pilsen, Pilsen, Czech Republic

**Keywords:** Renal cell carcinoma, Proton pump inhibitors, Statins, Antihypertensives, Acetyl salicylic acid, Beta-blockers, Drug-drug interactions, Immunotherapy, Nivolumab

## Abstract

Renal cell cancer (RCC) is typically a disease of older adults, who often have comorbidities requiring the use of multiple concomitant medications. Even though the patients with metastatic RCC (mRCC) are often exposed to concomitant medications in parallel with anticancer agents, the impact of such co-medications remains insufficiently explored. The aim of this study was to investigate the impact of the use of proton pump inhibitors (PPIs) and/or cardiovascular medication on the outcomes of patients with mRCC receiving nivolumab. Clinical data of patients with mRCC treated with nivolumab monotherapy were retrospectively analyzed with a focus on the association between progression-free survival (PFS) or overall survival (OS) and the use of common co-medications including PPIs, acetylsalicylic acid, statins, and antihypertensives. In total, 343 patients with mRCC were included. The median PFS and OS were 4.8 (95% CI 3.9–6.0) and 15.5 (95% CI 10.7–19.6) months vs. 9.7 (95% CI 7.6–12.2) and 29.8 (95% CI 23.7–33.1) months (*p* < 0.001 and *p* < 0.001) for PPI users and non-users, respectively. In the Cox multivariate analysis, the use of PPIs remained a significant factor predicting both inferior PFS (HR = 1.870 [95% CI 1.440–2.428], *p <* 0.001) and OS (HR = 1.674 [95% CI 1.235–2.270], *p* = 0.001).). We did not find any impact of the basic classes of antihypertensive drugs, statins, or acetylsalicylic acid on the survival outcomes. Present study results demonstrate the negative impact of concomitant PPI use on PFS and OS, whereas neither statins nor antihypertensive medications had a significant impact on survival outcomes in patients with mRCC receiving nivolumab monotherapy.

## Background

In the recent years, the advent of immunotherapy based on immune checkpoint inhibitors (ICIs) targeting the programmed cell death protein-1 (PD1)/ligand-1 (PD-L1) pathway has revolutionized systemic treatment of renal cell carcinoma (RCC). ICIs are the cornerstone of the current systemic metastatic RCC (mRCC) therapy. Nivolumab, an anti-PD1 monoclonal antibody, is widely used in combination regimens for the first line or as a single agent for the second or higher lines of systemic therapy. Because RCC is typically a disease of older adults, patients often have comorbidities that require the use of multiple concomitant medications [1]. Proton pump inhibitors (PPIs) are among the most commonly prescribed drugs worldwide. Notably, in patients with cancer, PPIs are used to reduce gastrointestinal toxicity associated with anticancer treatment [2]. Cardiovascular diseases, including arterial hypertension, are among the most common comorbidities present in 30–50% of patients with mRCC [3, 4]. RCC patients are more prone to hypertension than the general population because most have undergone nephrectomy or partial nephrectomy and, most importantly, hypertension is a common side effect of tyrosine kinase inhibitors (TKIs) that remain a backbone of systemic therapy. In general, PPIs and cardiovascular medication represent the most common concomitant medications in patients with mRCC. There has been an increasing interest in drug interactions or potential anticancer effects of commonly used drugs in recent times. Several studies have suggested that concomitant medications, such as PPIs, corticosteroids, and antibiotics, may have an adverse impact on the efficacy of ICIs in patients with various malignancies [5–16]. The effect of concomitant cardiovascular medication has been studied with inconclusive results. Hypertension is associated with the efficacy of TKIs, and the need for antihypertensive medications may be positively associated with outcomes. Several studies have shown an association between the use of beta-blockers or statins and a superior outcome of patients with mRCC receiving first-line tyrosine kinase inhibitors (TKIs) as well as in those treated with ICIs [17–22]. Other studies suggest a positive effect of angiotensin system inhibitors on the outcome of patients treated with TKIs [23, 24]. Despite these efforts, the role of concomitant medication in oncological outcomes remains underexplored.

The present study aimed to investigate the impact of the use of PPIs and selected cardiovascular medication on outcomes of patients with mRCC receiving nivolumab monotherapy.

## Patients and methods

### Study design

Clinical data from patients with mRCC receiving nivolumab monotherapy in the second or higher line were retrospectively analyzed. We assessed the association of progression-free survival (PFS) and overall survival (OS) with the use of common co-medications including PPIs, acetylsalicylic acid, statins, and antihypertensives represented by beta-blockers, angiotensin-converting-enzyme inhibitors (ACEIs), angiotensin II receptor blockers, calcium channel blockers and diuretics. The clinical data were obtained from the national Renal Cell Carcinoma Information System II (RENIS II) registry, which provides retrospective anonymized data on baseline patient clinical characteristics as well as on previous therapies for mRCC, laboratory parameters, treatment course and outcomes (http://renis.registry.cz). Data on concomitant medications were extracted from the hospital information systems and added to the registry data. The RENIS II registry and the use of registry data for analysis were approved on October 28, 2019 (the number201928/52/MOU), by the Multicentre Ethics Committee of the Masaryk Memorial Cancer Institute in Brno, Czech Republic. The informed consent was signed by all the patients included in the registry. Data from seven cancer centers in the Czech Republic were used in the present analysis.

### Patients and treatment

Nivolumab was administered intravenously as a single agent using one of the standard approved schedules (240 mg every two weeks or 480 mg every four weeks). The treatment was continued until disease progression, unacceptable toxicity, or patient refusal. None of the patients had received prior ICI therapy. The concomitant medication was assessed at the initiation of the nivolumab treatment.

### Outcome assessment

The follow-up visits including physical examination and routine laboratory tests were performed every two to four weeks, and computed tomography (CT) was performed every three to four months during the treatment. The tumor response for the PFS endpoint was assessed locally using Response Evaluation Criteria in Solid Tumors (RECIST) version 1.1 [25].

### Statistical analysis

Standard descriptive statistics and frequencies were used to characterize the patient cohort. Baseline patient characteristics listed in Table 1 were compared between PPI users and non-users using Pearson’s Chi-Square test, Fisher’s exact test, and Mann-Whitney U test. PFS was calculated as the interval between the treatment initiation date and the documented progression or death date. OS was defined from the date of treatment initiation until death. Patients without recorded progression or death were censored at the date of the last follow-up. PFS and OS were estimated using the Kaplan-Meier method (with linear interpolation to set calculation points), and all point estimates were accompanied by two-sided 95% confidence intervals. Median follow-up was calculated using the inverse Kaplan-Meier method. A univariable Cox proportional hazards model was used with the Kaplan-Meier method and Gehan-Wilcoxon significance test to assess associations of PFS or OS with co-medication. A stratified Cox model (allowing for different baseline hazards in various groups while assuming the same effect of the examined variable in all) was then used to adjust the univariable model for potential categorical confounders. A multivariable Cox proportional hazards model was then used to evaluate the effects and mutual independence of all potential prognostic factors. Hazard ratio (HR) estimates provided by the Cox models are presented with 95% confidence intervals. The level of statistical significance was set at α = 0.05, and all reported *p*-values are two-tailed. Statistical analysis was performed using Statistica (Version 12; StatSoft, Inc., TuIsa, OK, USA), MATLAB (R2021a, The MathWorks Inc., Natick, MA, USA), and SISA (Simple Interactive Statistical Analysis, https://www.quantitativeskills.com/sisa/).

**Table 1:** Baseline patient characteristics

Characteristic, *n*(%)	Overall	Use of proton pump inhibitors (PPIs)		*p*-value
		No	Yes	
	*n* = 343	*n* = 232	*n* = 111	
Gender, n (%)				0.841
Male	251 (73.2)	169 (72.8)	82 (73.9)	
Female	92 (26.8)	63 (27.2)	29 (26.1)	
Age at treatment initiation (years)				0.346
Median (range)	68.0 (39.2–87.9)	68.2 (39.2–87.5)	67.5 (42.2–87.9)	
ECOG PS, n (%)				**< 0.001**
0	106 (33.9)	84 (40.2)	22 (21,2)	
1	205 (65.5)	125 (59.8)	80 (76,9)	
2	2 (0.6)	0 (0)	2 (1,9)	
Unknown	30	-	-	
Histology, n (%)				1.000
Clear cell carcinoma	326 (95.3)	220 (95.2)	106 (95.5)	
Other	16 (4.7)	11 (4.8)	5 (4.5)	
Unknown	1	-	-	
Sarcomatoid or rhabdoid component, n (%)				0.6245
Yes	21 (7.8)	16 (8.6)	5 (6.1)	
No	248 (92.2)	171 (91.4)	77 (93.9)	
Unknown	74	-	-	
Primary tumor grade, n (%)				0.185
G1-2	131 (47.0)	93 (49.7)	38 (41.3)	
G3-4	148 (53.0)	94 (50.3)	54 (58.7)	
Unknown	64	-	-	
MSKCC risk group, n (%)				0.326
Favorable-risk group	80 (32.5)	57 (35.4)	23 (27.1)	
Intermediate-risk group	155 (63.0)	96 (59.6)	59 (69.4)	
Poor	11 (4.5)	8 (5.0)	3 (3.5)	
Unknown	97	-	-	
Synchronous metastases, n (%)				**0.028**
Yes	135 (39.4)	82 (35.3)	53 (47.7)	
No	208 (60.6)	150 (64.7)	58 (52.3)	
Line of systemic therapy, n (%)				0.920
Second	215 (62.7)	145 (62.5)	70 (63.1)	
Third or higher	128 (37.3)	87 (37.5)	41 (36.9)	
Previous nephrectomy, n (%)				0.139
Yes	281 (81.9)	195 (84.1)	86 (77.5)	
No	62 (18.1)	37 (15.9)	25 (22.5)	
Bone metastases, n (%)				0.102
Yes	55 (16.0)	32 (13.8)	23 (20.7)	
No	288 (84.0)	200 (86.2)	88 (79.3)	
Year of treatment initiation, n (%)				0.8733
Before 2020	174 (50.7)	117 (67.2)	57 (32.8)	
After 2020	169 (49.3)	115 (68.0)	54 (32.0)	
Type of PPI, n (%)				-
Omeprazol	-	-	85 (76.6)	
Pantoprazol	-	-	19 (17.1)	
Esomeprazol	-	-	4 (3.6)	
Loseprazol	-	-	1 (0.9)	
Lanzoprazol	-	-	2 (1.8)	
Daily PPI dose, n (%)				-
≤ 20 mg	-	-	73 (68.2)	
> 20 mg	-	-	34 (31.8)	

ECOG PS = Eastern Cooperative Oncology Group Performance Status; CI = Confidence interval; MSKCC = Memorial Sloan Kettering Cancer Center; PPI = proton pump inhibitor

## Results

### Patient characteristics

The study cohort included 343 consecutive patients with mRCC receiving nivolumab monotherapy as the second or subsequent line of systemic therapy between 2013 and 2023. At the time of nivolumab therapy initiation, 111/343 (32.4%) patients were using PPIs, 58/342 (17.0%) patients were using acetylsalicylic acid, 95/337 (28.2%) patients were using statins, 102/340 (30.0%) patients were using beta-blockers, 122/343 (35.6%) patients were using ACEIs, 41/343 (12.0%) patients were using angiotensin II receptor blockers, 129/343 (37.6%) patients were using calcium channel blockers and 84/343 (29.1%) patients were using diuretics. The baseline patient characteristics are summarized in Table 1.

The cohort of PPI users included more patients with higher Eastern Cooperative Oncology Group performance status (ECOG PS) and those with synchronous metastases (*p* < 0.001 and *p* = 0.028, respectively; see Table 1). Patients using statins and beta-blockers were significantly older compared to others (median age 71.5 vs. 66.5 years, *p* < 0.001, and 69.1 vs. 67.6 years, *p* = 0.043, respectively).

### Outcome of patients according to the use of concomitant medications

At the time of data analysis, 234 (68.2%) patients progressed on nivolumab, 198 (57.7%) patients died, and the median follow-up time was 33.7 months. Median PFS and OS for the cohort were 7.6 months (95% CI 6.4–9.6) and 23.6 months (95% CI 20.2–29.4), respectively.

The univariate Cox analysis evaluating the impact of the assessed concomitant medications on patient survival revealed that PPIs were significantly associated with inferior PFS and OS (HR = 1.809 [95% CI 1.409–2.323], *p* < 0.001 and HR = 1.698 [95% CI 1.265–2.277], *p* < 0.001, respectively) (Table 2).

**Table 2:** Univariate Cox-proportional hazards model assessing the impact of the used concomitant medication on progression-free survival (PFS) and overall survival (OS)

Concomitant medication	Progression-free survival (PFS)		Overall survival (OS)	
	HR (95% CI)	*p*-value	HR (95% CI)	*p*-value
Proton pump inhibitors (PPIs)	1.809 (1.409–2.323)	**< 0.001**	1.698 (1.265–2.277)	**< 0.001**
Acetylsalicylic acid	0.824 (0.591–1.149)	0.254	1.029 (0.708–1.496)	0.879
Statins	0.830 (0.634–1.086)	0.175	0.990 (0.723–1.356)	0.951
Beta-blockers	0.905 (0.693–1.182)	0.465	0.869 (0.630–1.199)	0.393
Angiotensin-converting-enzyme inhibitors (ACEIs)	0.804 (0.624–1.035)	0.091	0.766 (0.566–1.036)	0.083
Angiotensin receptor blockers	0.888 (0.614–1.285)	0.529	1.082 (0.716–1.635)	0.707
Calcium channel blockers	0.949 (0.741–1.216)	0.680	0.837 (0.623–1.124)	0.236
Diuretics	0.932 (0.699–1.243)	0.631	0.917 (0.651–1.293)	0.621

HR = hazard ratio; CI = Confidence interval

**Fig. 1:**
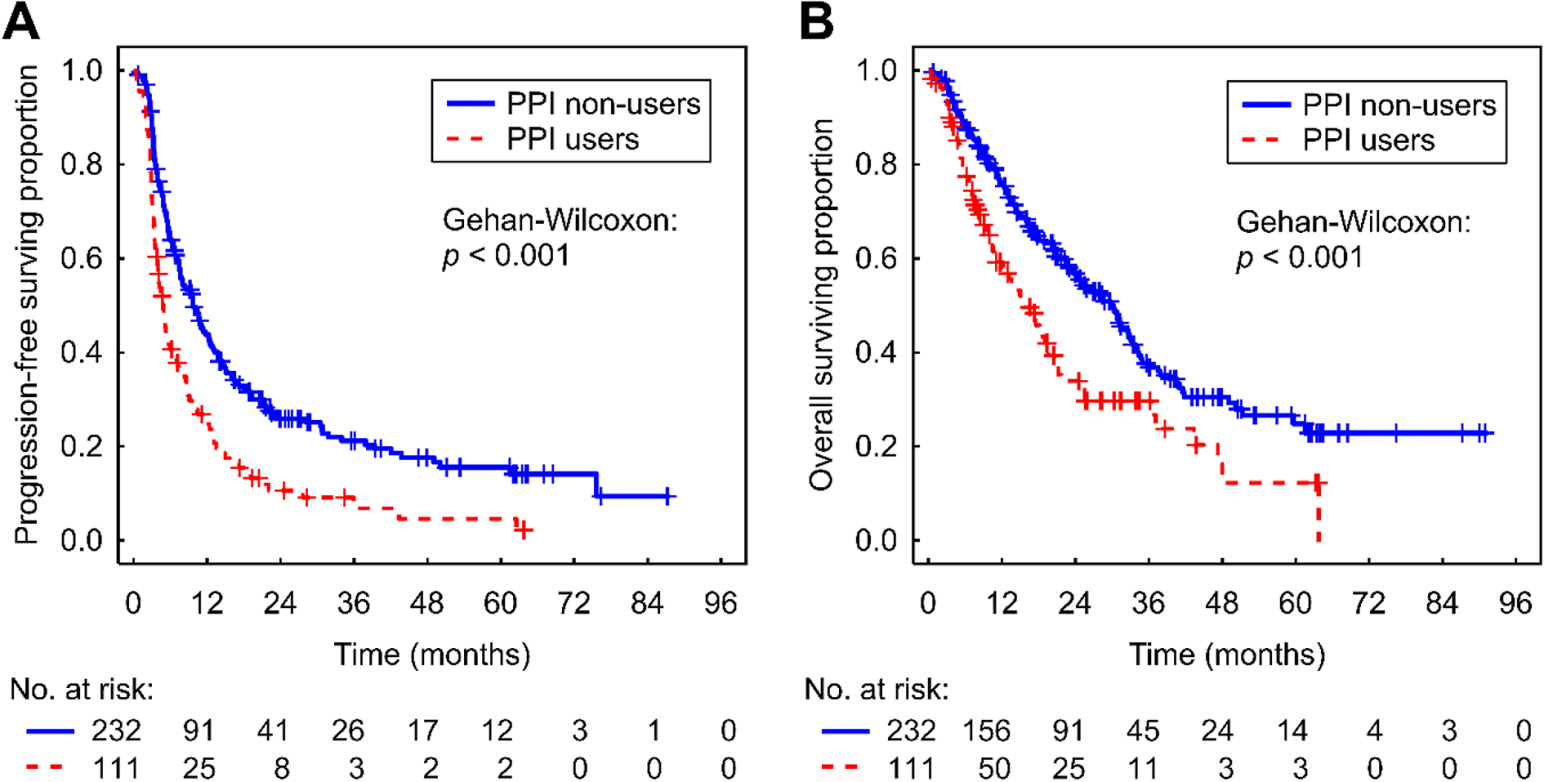
Kaplan-Meier estimates of progression-free survival (PFS) (**A**) and overall survival (OS) (**B**) according to the concomitant use of proton pump inhibitors (PPIs)

The median PFS and OS for patients using PPIs was 4.8 months (95% CI 3.9–6.0) and 15.5 (95% CI 10.7–19.6) months compared to 9.7 (95% CI 7.6–12.2) and 29.8 (95% CI 23.7–33.1) months for those not using PPIs (*p* < 0.001 and *p* < 0.001, respectively) (Table 3; Fig. 1). Even when adjusted for the potentially confounding effect of ECOG PS using a stratified Cox model, the difference in PFS (HR = 1.703 [95% CI 1.308–2.218]) and OS (HR = 1.568 [95% CI 1.151–2.136]) remained statistically significant (*p* < 0.001 and *p =* 0.004, respectively).

**Table 3:** Progression-free survival (PFS) and overall survival (OS) according to the use of proton pump inhibitors (PPIs)

Survival	Use of proton pump inhibitors (PPIs)		*p*-value
	No	Yes	
Median PFS (CI 95%)	9.7 months (7.6–12.2)	4.8 months (3.9–6.0)	< 0.001*
3 months PFS (CI 95%)	87.8% (83.5–92.0)	72.7% (64.4–81.0)	
6 months PFS (CI 95%)	63.8% (57.6–70.1)	40.6% (31.4–49.9)	
12 months PFS (CI 95%)	43.6% (37.1–50.2)	25.2% (16.9–33.5)	
Median OS (CI 95%)	29.8 (23.7–33.1)	15.5 (10.7–19.6)	< 0.001*
12 months OS (CI 95%)	75.7% (70.0–81.4)	58.2% (48.4–68.0)	
18 months OS (CI 95%)	64.3% (57.8–70.8)	44.8% (34.5–55.1)	
24 months OS (CI 95%)	56.5% (49.6–63.4)	33.0% (22.9–43.2)	

PFS = progression-free survival; OS = overal survival; CI = Confidence interval

* The *p*-value characterizes survival comparison over the whole follow up period, i.e. it is valid for median as well as all fixed-timepoint estimates

In the multivariate Cox analysis, including the whole range of potential prognostic variables, the use of PPIs remained a significant factor in predicting inferior outcomes concerning both PFS (HR = 1.870 [95% CI 1.440–2.428], *p <* 0.001) and OS (HR = 1.674 [95% CI 1.235–2.270], *p* = 0.001). OS was also inferior with ECOG PS of 1/2 (HR = 1.472 [95% CI 1.045–2.074], *p* = 0.027) and in intermediate/poor-risk group according to MSKCC (HR = 1.648 [95% CI 1.072–2.533], *p* = 0.023), while previous nephrectomy was associated with improved OS (HR = 0.624 [95% CI 0.426–0.913], *p* = 0.015). PFS was inferior with sarcomatoid or rhabdoid differentiation (HR = 1.724 [95% CI 1.028–2.889], *p* = 0.039). The results of the multivariate Cox analysis are summarized in Table 4.

**Table 4:** Multivariate Cox-proportional hazards model for progression-free survival (PFS) and overall survival (OS)

Parameter	Category	PFS		OS	
		HR (95% CI)	*p*-value	HR (95% CI)	*p*-value
Gender			0.155		0.447
	Female	1		1	
	Male	0.820 (0.624–1.078)		1.140 (0.814–1.595)	
Age			0.716		0.827
	< 70 years	1		1	
	≥ 70 years	0.954 (0.741–1.229)		1.033 (0.772–1.383)	
ECOG PS			0.137		**0.027**
	0	1		1	
	1 or 2	1.247 (0.932–1.667)		1.472 (1.045–2.074)	
Line of therapy			0.929		0.450
	Second	1		1	
	Third or higher	1.012 (0.775–1.322)		0.887 (0.650–1.211)	
Histology			0.840		0.177
	Clear cell carcinoma	1		1	
	Other	1.065 (0.577–1.967)		1.551 (0.820–2.933)	
Sarcomatoid or rhabdoid differentiation			**0.039**		0.182
	No	1		1	
	Yes	1.724 (1.028–2.889)		1.494 (0.829–2.693)	
Grade			0.784		0.286
	G1-G2	1		1	
	G3-G4	0.961 (0.723–1.277)		0.833 (0.595–1.166)	
Synchronous metastases			0.607		0.325
	No	1		1	
	Yes	1.079 (0.808–1.441)		1.190 (0.842–1.682)	
MSKCC risk group			0.906		**0.023**
	Favorable-risk group	1		1	
	Intermediate or poor-risk group	1.020 (0.735–1.415)		1.648 (1.072–2.533)	
Previous nephrectomy			0.078		**0.015**
	No	1		1	
	Yes	0.730 (0.515–1.036)		0.624 (0.426–0.913)	
Year of treatment initiation			0.448		0.637
	Before 2020	1		1	
	After 2020	1.104 (0.855–1.427)		1.078 (0.789–1.472)	
Use of PPIs			**< 0.001**		**0.001**
	No	1		1	
	Yes	1.870 (1.440–2.428)		1.674 (1.235–2.270)	

PFS = progression-free survival; OS = overal survival; HR = hazard ratio; CI = Confidence interval; HR = hazard ratio; PPIs = proton pump inhibitors; ECOG PS = Eastern Cooperative Oncology Group Performance Status; MSKCC = Memorial Sloan Kettering Cancer Center

Additionally, when the impact of daily PPI dose was analyzed, no significant effect on PFS (*p* = 0.836) or OS (0.658) was found between patients using ≤ 20 mg compared to those using > 20 mg daily.

No significant association was detected between patients’ outcomes and the use of other assessed concomitant medications, including beta-blockers, ACEIs, angiotensin II receptor blockers, calcium channel blockers, diuretics, acetylsalicylic acid, and statins.

## Discussion

The results of the present retrospective study suggest that the concomitant use of PPIs is associated with inferior PFS and OS in patients with mRCC treated with nivolumab monotherapy, while no effect of daily PPI dose was seen. We did not find any significant association between patients’ outcomes and the use of other concomitant medications assessed, including beta-blockers, ACEIs, angiotensin II receptor blockers, calcium channel blockers, diuretics, acetylsalicylic acid, and statins. The multivariable Cox proportional hazards model confirmed that using PPIs was an independent risk factor for PFS and OS.

Several hypotheses may be proposed to explain the association between PPI use and outcome in patients with mRCC, including the alterations of gut microbiome, effects on the immune system or an association of PPI with other conditions or medications affecting the patient outcome. The gut microbiome widely interacts with the immune system and significantly affects the tumor microenvironment, which can vary from one individual to another. The microbiome’s vital role and its composition’s impact on the response to immunotherapy have been demonstrated in multiple experimental and clinical studies. It has been suggested that the microbiome plays a crucial role in the efficacy of ICIs. However, the specific mechanisms are not fully understood. The composition of the human gut microbiome is affected by various intrinsic and extrinsic factors, including diet, alcohol consumption, lifestyle, host genetics, and multiple medications. PPIs and antibiotics are among drugs with a well-known effect on the gut microbiome. PPIs directly affect the gut microbiota by altering gastric pH and delaying gastric emptying rate [26, 27]. Two large studies conducted by Imhann et al. and Jackson et al. found a significant decrease in alpha diversity and changes in bacterial species, leading to a higher abundance of oral commensals in the population of PPI users compared with non-users [28, 29]. Specifically, the bacterial taxa associated with response to ICIs, including *Ruminococcaceae*, *Bifidobacterium sp.*, *Alistipes sp.*, and *Akkermansia muciniphila*, were reported to be decreased. In contrast, the bacteria associated with resistance to ICIs, including *Bacteroidetes* and *Escherichia coli*, were reported to be increased in PPI users [28, 30–33]. Thus, the ability of PPIs to influence the effect of ICIs may be primarily related to the changes in the gut microbiome composition, which has been suggested to play an essential role in patients treated with ICIs for advanced solid cancers [34]. Additionally, it has been suggested that PPIs may directly disrupt immune system function by suppressing the expression of adhesion molecules, and in vitro studies also show a detrimental effect on neutrophil functions and cytotoxic activity of natural killer cells, both potentially contributing to decreased effectivity of ICIs in cancer treatment [35, 36].

There has been increasing evidence that the concomitant use of PPIs may have a detrimental effect on the outcome of ICI therapy in various types of cancer [5–16]. The findings of a meta-analysis recently conducted by Lopes et al., including 41 studies with a total of 20,042 patients, suggest that PPI treatment is associated with worse outcomes in advanced patients with cancer treated with ICIs in terms of PFS (HR = 1.28; 95%CI, 1.15–1.42) and OS (HR = 1.37; 95%CI, 1.23–1.52) [37]. The impact of PPIs on the outcome of patients with mRCC treated with ICIs has been insufficiently explored due to the limited and inconsistent data available. The most extensive retrospective study, analyzed 1,091 patients treated with ICIs for various cancers, mostly non-small cell lung cancer and melanoma. PPIs were associated with worse OS, but interestingly the association was not detected in patients receiving recent chemotherapy. The authors suggested that the disruptive effect of chemotherapy on gut microbiome may override the effect of PPIs [38].

Another large retrospective study including 1,012 patients with cancer patients (185 patients with mRCC), showed that PPIs have an adverse prognostic effect on PFS (HR 1.26; 95% CI 1.07–1.48) and OS (HR 1.26; 95% CI 1.04–1.52); however, the data for the mRCC subgroup was not shown separately [39]. In agreement with the present results, Ugrakli et al. have recently reported the findings from a retrospective study showing that concomitant use of PPIs was significantly associated with worse PFS (6.4 vs. 9.7 months, *p* = 0.03) and also OS (14.6 vs. 29.9 months, *p* = 0.01) in 109 patients with mRCC receiving second-line nivolumab therapy (50 PPI users vs. 59 PPI non-users). The use of PPI was found to be an independent risk factor for inferior PFS (non-use vs. use, HR: 0.44, 95%Cl 0.28–0.96, *p* = 0.014) and OS (non-use vs. use, HR: 0.68, 95%Cl 0.22–0.88, *p* = 0.01), respectively [40]. On the other hand, contradictory results showing no impact of concomitant PPI use on the outcome of patients with mRCC treated with nivolumab have been reported by Mollica et al. and Rassy et al. [41, 42]. The retrospective study conducted by Mollica et al. included 218 patients with mRCC who received the combination of ipilimumab and nivolumab for first-line treatment (62 patients, 25 PPI users vs. 37 PPI non-users) or nivolumab monotherapy for second or third line treatment (156 patients, 88 PPI users vs. 68 PPI non-users). Despite not demonstrating any significant impact of the concomitant PPI use on the overall response rate (ORR) or survival outcomes, the reported results indicate numerically more prolonged survival for PPI non-users, particularly in the subgroup treated with nivolumab monotherapy (PFS: 12.2 vs. 8.5 months, *p* = 0.928; OS: not reached vs. 27.3 months, *p* = 0.84) [41]. This study was limited by a relatively low number of patients, particularly those in the specified subgroups. Rassy et al. conducted a retrospective analysis of prospectively collected data from the GETUG-AFU 26 NIVOREN phase II trial, including 707 patients with mRCC (196 PPI users vs. 511 PPI non-users) receiving nivolumab in the second or higher line of systemic therapy. Similarly, the authors reported no significant impact of concomitant PPI use on survival outcomes in the multivariable analysis after adjusting for potentially confounding factors (PFS: HR = 0.89, 95% CI 0.74–1.08; OS: HR = 1.24; 95% CI, 0.98–1.58). Despite the negative results of the multivariable analysis, a more prolonged OS for PPI non-users compared to PPI users (17.7 vs. 24.7 months, HR = 1.41, *p* = 0.003) was found [42]. Notably, a significant imbalance in several prognostic factors between the groups of PPI users and non-users in this study should be pointed out, introducing a potential bias. The group of PPI users included significantly more patients with poor risk (34.9% vs. 22.4%) and fewer patients with favorable risk (10.3% vs. 21.0%) according to IMDC prognostic model (*p* < 0.001), more patients with ECOG PS 0-2-3 (21.2% vs. 13.2%) (*p* = 0.011) [42]. Aside from immunotherapy, the negative effects of PPI use on the outcome of patients with mRCC were demonstrated in two studies that focused on the role of concomitant medications in mRCC treated with TKIs [43, 44]. On the contrary, our previous results do not support these findings [17]. The rate of patients taking PPI differed markedly among the studies, and it is possible that the conditions or other medications associated with PPI use contributed to the reported differences in outcomes.

Regarding the impact of concomitant antihypertensive medication in patients with mRCC, positive results have been previously reported for beta-blockers or angiotensin system inhibitors in patients receiving TKIs [17, 18, 23, 24]. Of note, there is a lack of data on patients with mRCC treated with ICIs, despite the frequent use of concomitant cardiovascular medication in this patient population. Patel et al. reported superior outcomes for patients using concurrent beta-blockers in a small retrospective study including 48 patients with metastatic urothelial cancer or mRCC that received ICIs after prior chemotherapy or targeted therapy (OS: not reached vs. 11.6 months, *p* = 0.004) [19]. These results must be interpreted cautiously due to the very limited number of patients, particularly those with mRCC (14 patients included) [19]. In the present study, we found no significant association between the use of specific antihypertensive types and survival outcomes.

Statins have long deserved a particular interest in patients with cancer because of their potential anticancer properties based on experimental studies [45]. Several, mostly retrospective, studies have been conducted with inconclusive results. The data from meta-analyses by Luo et al. and Wu et al. did not show significant effects of statins on the outcome of RCC patients [46, 47]. On the contrary, the results from two other meta-analyses conducted by McKay et al. and Nayan et al., and most recently, by Liang et al. suggested that the use of statins may be associated with improved survival in patients with RCC [20–22]. The present study did not observe a significant association between concomitant statin use and survival outcomes. Thus, our results do not support previous findings from a retrospective study conducted by Santoni et al., showing a significant impact of statins on OS (34.4 vs. 18.6 months, *p* = 0.017) and PFS (11.7 vs. 4.6 months, *p* = 0.013) in 219 patients with mRCC (59 statin users vs. 160 statin non-users) receiving nivolumab monotherapy. Of note, a previous study by our group showed no impact of statins in patients with mRCC treated with TKIs [48]. Although the association between antibiotic use and response to immunotherapy is supported by existing evidence, our study lacks data on antibiotic use before or during nivolumab treatment, which limits our ability to assess its potential influence on this association.

Present study has several limitations, primarily because of its retrospective design. The duration of the investigated concomitant medication exposure could not be assessed from the available data sources. There has not been a centralized review of the radiology imaging results. The group of PPI non-users included more patients with better performance status (ECOG PS 0) and those with metachronous metastases compared to PPI users creating an imbalance in baseline characteristics, although the estimation of PFS and OS differences was adjusted for ECOG PS and both of these parameters were also included in the multivariable Cox model. However, the inability to identify an association with any of the other drug classes could also be viewed as an internal control for matching the patient groups. The study was probably underpowered to detect differences for some of the subgroups. The data collected did not allow for the analysis of the conditions or, possibly, other medications not captured by this survey, leading to PPI use, and it is possible that condition that led to PPI use, e.g. prior corticosteroid therapy, could be responsible for inferior outcomes with nivolumab. PPI use might simply be a marker of a patient population at higher risk for inferior outcomes, as suggested by the association with poor ECOG PS. However, the fact that the use of PPIs kept its significance in the multivariable Cox model proves that it contains new prognostic information on top of that already provided by ECOG PS. Moreover, in studies analyzing the relation between concomitant medication and survival outcomes, a potential positive effect of a given medication may be neutralized by the negative effect on survival of comorbid condition being treated. In RCC, these interactions may be even more complex as nephrectomy, which is associated with improved survival outcomes, may lead to higher risk of hypertension and the need for antihypertensive medications. The need for hypertensive medication during TKI treatment has been shown to be associated with improved outcomes, yet in the long-term any cardiovascular event would neutralize this benefit [49]. In the present study, nivolumab was used as monotherapy after failure of TKIs. While this approach was a standard of subsequent treatment during the initial introduction of nivolumab, the current standard of care for most patients with mRCC includes first-line immune-combination regimens. Therefore, further research is needed to elucidate the impact of PPI use in these patients.

## Conclusions

The results of the present retrospective multicenter study demonstrate a significant impact of concomitant PPI use on PFS and OS. In contrast, neither statins nor specific antihypertensive medications had a significant effect on the outcomes of patients with mRCC receiving nivolumab monotherapy. Further research into the clinical, biological and immunological basis of this interplay, may be needed to optimize the outcomes for patients with mRCC treated with immunotherapy.

## Data Availability

The datasets generated during and/or analyzed during the current study are available from the corresponding author on reasonable request.
